# The Impact of Liver Transplantation after Surgical Treatment of Hepatocellular Carcinoma

**DOI:** 10.3389/fsurg.2014.00029

**Published:** 2014-07-22

**Authors:** Halit Topal, Joyce Tiek, Steffen Fieuws, Jacques Pirenne, Frederik Nevens, Baki Topal

**Affiliations:** ^1^Department of Abdominal Surgery, University Hospitals Leuven, Leuven, Belgium; ^2^Department of Biostatistics, I-Biostat, Katholieke Universiteit Leuven en Hasselt, Leuven, Belgium; ^3^Department of Transplantation Surgery, University Hospitals Leuven, Leuven, Belgium; ^4^Department of Hepatology, University Hospitals Leuven, Leuven, Belgium

**Keywords:** hepatocellular carcinoma, surgery, transplantation, survival

## Abstract

**Background:** The impact of liver transplantation (LTx) after surgical treatment for hepatocellular carcinoma (HCC) remains undefined. The aim of the current study was to assess the impact of LTx and of selection criteria for LTx on the survival of patients who underwent surgery for HCC.

**Methods:** Between 2004 and 2009, 119 patients underwent surgical treatment for HCC. Cirrhosis was present in 85 patients. Of all patients, 77 fulfilled the Milan criteria, 88 the UCSF and 87 the up-to-7 criteria. Finally, 35 patients received an LTx, of whom 31 met the Milan, 33 the UCSF, and 33 the up-to-7 criteria. The relation between LTx and survival was evaluated using a Cox regression model with LTx as a time-dependent factor.

**Results:** Median [95% confidence interval (CI)] disease-free survival (DFS) and overall survival (OS) of the entire patient population was 9.4 (7–12.2) and 49.1 (37.7–64) months, respectively. The 1, 3, and 5-year DFS vs. OS rates were 36, 3, and 0% vs. 84.7, 61.7, and 39.6%, respectively. Patients fulfilling the Milan criteria had a significantly better OS and DFS than those who had tumors beyond the Milan criteria (*p* < 0.047). No significant differences were observed in terms of OS between patients within vs. beyond the UCSF or up-to-7 criteria (*p* > 0.130). In multivariable analysis, cirrhotic patients who received an LTx had a better OS, with a hazard ratio equal to 0.25 (95% CI: 0.08–0.74; *p* < 0.01). LTx after surgery had a beneficial impact on both DFS and OS of patients in all the three selection criteria models of LTx (*p* < 0.031).

**Conclusion:** LTx after primary surgery seems to offer the best long-term survival for patients suffering from HCC in cirrhosis as well as for them who fulfill the Milan, UCSF, and up-to-7 criteria.

## Background

Hepatocellular carcinoma (HCC) is one of the most common and aggressive malignancies worldwide (1). The vast majority of patients with HCC have an underlying chronic liver disease, often at the stage of cirrhosis. Factors responsible for the poor prognosis include late onset diagnosis, underlying cirrhosis, and resistance to chemotherapy. Despite current improvements in treatment and diagnostics, only 30–40% of patients with HCC are eligible for curative treatment options that include liver transplantation (LTx), surgical resection, and local ablative therapies (LAT) (2–4).

To date, no randomized studies exist to compare results of these therapeutic options and, therefore, no definite conclusion can be drawn about which treatment offers the best outcome. In patients with well-preserved liver function, surgical resection is currently the standard of care for early-stage HCC (5, 6). Several minimally invasive options can be offered to patients who are not eligible for surgical resection. One of the most effective LAT is radiofrequency ablation (RFA), which is now considered potentially curative for selected patients with early-stage small HCC (7–9). On the other hand, LTx is a suitable option for early HCC in cirrhotic patients, though organ shortage remains a challenge hard to overcome. The Milan criteria are most widely used to select candidates for LTx, whereas the UCSF and up-to-7 criteria consider patients with intermediate-stage tumors and those exceeding the Milan criteria (10–12).

In the absence of well-designed randomized studies addressing the best therapeutic modality, the impact of bridging therapy before LTx as well as that of LTx after surgical treatment for HCC remains undefined. The aim of the current study was to assess the impact of LTx and of selection criteria for LTx on the survival of patients who underwent surgical treatment for HCC.

## Methods

### Patient population

From January 2004 until April 2009, clinical data of 135 consecutive patients who underwent surgical treatment for HCC were collected prospectively. The study was approved by the KU Leuven ethical committee prior to patient recruitment and received the study number ML1295. The study was carried out in compliance with the Helsinki declaration and written informed consent was obtained from participants. Patients, who underwent their primary liver surgery in another institution and were referred for repeat hepatic surgery, were excluded from the study (*n* = 16). Thus, 119 patients [male/female ratio 85/34; median (range) age 66 years (27–88 years) were considered for further analysis]. Liver resection (LR) was performed in 53 patients, RFA in 58, and LR + RFA in 8 patients.

Cirrhosis was present in 85 patients with a Child-Pugh classification A in 65 patients, B in 19, and C in 1 patient. The median (range) model for end-stage liver disease (MELD) score was 9 (6–18). Pre-operative hepatic transarterial chemo-embolization (TACE) with doxorubicin was performed in 14 patients within 3 months before liver surgery. The median serum alpha-fetoprotein (AFP) level prior to surgery was 9.9 μg/L (0.7–113,300) while 18 patients had a level above 400 μg/L. At the time of surgery, benign ascites was observed in 14 patients. Liver fibrosis, confirmed on histopathology examination, was present in 11 patients.

Of the entire study population, 77 patients fulfilled the Milan criteria, 88 the UCSF, and 87 the up-to-7 criteria. Finally, 35 patients received an LTx at a median time interval of 7.3 (1.7–34.6) months after surgery. Of these patients, 31 met the Milan criteria, 33 the UCSF, and 33 the up-to-7 criteria.

### Surgical procedure

Laparoscopic liver surgery (LLS) (resection 13; RFA 54; resection + RFA 5) was performed in 72 patients for a total of 93 tumors (13 patients with 2 and 4 patients with 3 tumors). The median (range) of the maximum tumor diameter in the LLS-group was 26.5 mm (12–190). Open liver surgery (OLS) (resection 40; RFA 4; resection + RFA 3) was performed in 47 patients for a total of 55 tumors (8 patients with 2 tumors). The median of the maximum tumor diameter in the OLS-group was 70 mm (8–240). Tumor diameter and the resection/RFA ratio were higher in the OLS- vs. LLS-group (*p* < 0.001). RFA was considered for patients with HCC smaller than 3 cm in diameter, for unresectable HCC, and lesions in contact with major vascular structures to obtain tumor-free resection margins or result in too small liver remnant. Major liver resection (>3 segments) was performed in 26 and minor (<2 segments) resection in 35 patients.

### Follow-up

Patient follow-up was closed in September 2010, with a median follow-up time after surgery of 29.8 (range 0–75.1) months (including deceased patients). Follow-up information was obtained through review of patients’ hospital charts that were prospectively registered in our institution’s database. Postoperative follow-up investigations consisted of a clinical examination, biochemistry including serum AFP level, magnetic resonance imaging (MRI) scan of the liver, and a contrast-enhanced computed tomography (CT) scan of thorax performed every 3–4 months. Recurrent disease was defined as a radiological mass characterized as a malignant lesion on MRI- and/or CT-scan.

### Outcome measures and predictive factors

Postoperative complications were classified based on the therapy-oriented severity grading system (TOSGS) and allocated to surgical site (SSC) vs. non-surgical site complications (NSSC) (13). Overall survival (OS) was defined as time from surgery to death, irrespective of cause. Disease-free survival (DFS) was defined as time to tumor recurrence or death, irrespective of cause. Cox regression analyses were performed to assess the prognostic value of 18 potential predictive factors, i.e., age, gender, American Society of Anesthesiologists (ASA) score, serum AFP level, number of tumors, maximum tumor diameter, Child-Pugh score, MELD score, location of HCC (right/left liver), resection, RFA, extent of liver resection (major or minor), pre-operative TACE, presence of intra-operative ascites, cirrhosis, liver fibrosis, and LTx after surgery.

### Statistical analysis

Mann–Whitney *U* and Fisher Exact tests were used to compare patient variables between two groups. Cumulative incidence estimates were used to construct a curve for time to liver transplant (TLTx), considering death without an LTx as a competing event. Pepe and Mori’s test was used to compare this curve between MILS and OLS. Log-rank tests and Cox regression models were used to verify the relation between the set of predictors and TLTx, OS, and DFS, respectively. For OS and DFS, patients lost to follow-up were censored. In the analysis of TLTx, deaths without LTx were also censored. The proportional hazards assumption and the functional form of the continuous predictors were verified by applying graphical and numerical methods.

Kaplan–Meier estimates were used to construct curves for OS and DFS, which were compared between groups using a log-rank test. Since the moment of LTx differed between patients, the relation between LTx and survival was evaluated using a Cox regression model with LTx as a (irreversible) time-dependent factor. Treating LTx as a time-varying factor implies that at the moment of surgery all patients belong to the no-transplant group; when a patient receives an LTx he/she switches to the transplant group.

*P*-values that are smaller than 0.05 were considered significant. Analyses were performed using SAS software, version 9.2 of the SAS System for Windows (Copyright©2002 SAS Institute Inc., SAS and all other SAS Institute Inc., product or service names are registered trademarks or trademarks of SAS Institute Inc., Cary, NC, USA).

## Results

### Postoperative outcome

Postoperative complications were observed in 22 (18.5%) patients. According to the TOSGS score, the severity of postoperative complications were grade 1 in 2, grade 2 in 6, grade 3b in 5, grade 4a in 4, grade 4b in 1, and grade 5 or death in 4 patients. Complications were allocated to SSC in 8 and NSSC in 14 patients. The median postoperative length of hospital stay (LOS) was 6 days (range 1–27; IQR 3–10).

### Long-term survival

The median [95% confidence interval (CI)] DFS and OS of the entire patient population was 9.4 (7–12.2) and 49.1 (37.7–64) months, respectively. The 1, 3, and 5-year DFS vs. OS rates were 36, 3, and 0% vs. 84.7, 61.7, and 39.6%, respectively.

Patients fulfilling the Milan criteria had a significantly better OS and DFS than those who had tumors beyond the Milan criteria (*p* < 0.047) (Table 1). Disease-free survival rates were better in patients within both the UCSF (*p* = 0.006) and the up-to-7 (*p* = 0.007) criteria as compared to that of patients beyond these criteria. No significant differences were observed in terms of OS between patients within vs. beyond the UCSF or up-to-7 criteria (*p* > 0.130) (Table 1).

**Table 1:** **Survival after surgery for HCC in patients as classified according to criteria for liver transplant**.

Criteria for liver transplant	Disease-free survival						Overall survival				
		Median (95% CI); months	1 years (%)	3 years (%)	5 years (%)	Log-rank *p*-value	Median (range); months	1 years (%)	3 years (%)	5 years (%)	Log-rank *p*-value
Milan	Yes (*n* 77)	35.2 (25.3-ND)	67.4	45.5	42.8	0.016	53.0 (39.4-ND)	88.1	64.7	47.1	0.047
	No (*n* 42)	14.6 (7.8–21.2)	53.9	28.6	19.1		39.4 (24.0–58.8)	76.1	52.5	26.2	
UCSF	Yes (*n* 88)	31.7 (20.6-ND)	69.9	44.0	41.5	0.006	49.1 (39.4–68.4)	86.1	64.8	42.7	0.237
	No (*n* 31)	10.8 (5.6–26.4)	45.8	26.6	13.3		39.4 (14.9-ND)	77.3	52.0	28.9	
Up-to-7	Yes (*n* 87)	31.7 (19.7-ND)	69.9	44.0	41.5	0.007	53.0 (39.4–68.4)	88.3	65.6	43.2	0.130
	No (*n* 32)	12.2 (5.6–26.4)	46.3	27.0	13.5		39.4 (12.4-ND)	71.6	50.9	28.3	

**Figure 1:**
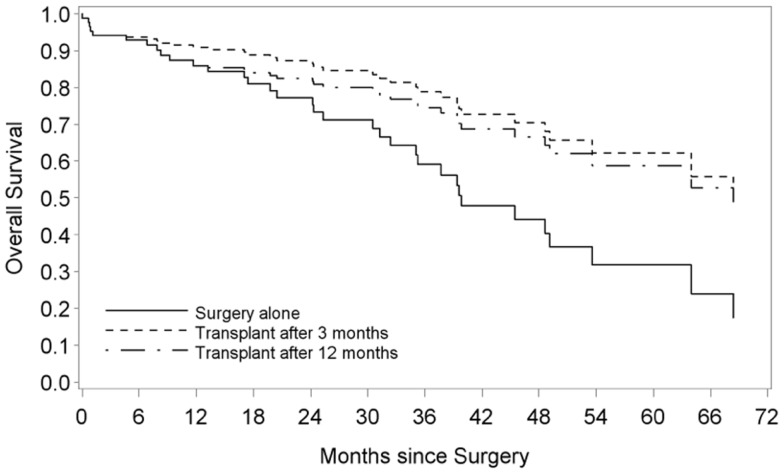
**Overall survival of cirrhotic patients with HCC after surgery and liver transplantation vs. surgery alone**. Survival curves are obtained from a Cox regression with liver transplant as a time-dependent variable. The solid line represents patients without a liver transplant. The non-solid lines represent hypothetical samples of patients who receive a liver transplant after 3 and 12 months, respectively.

Median OS of patients who met the Milan criteria and who underwent LTx after surgery was 64 months (CI 45.5 – undetermined) as compared to 35.2 months (CI 24.2 – undetermined) in patients who were treated with surgery alone (*p* = 0.035) (Figure 1). The hazard ratio (HR) comparing the OS of cirrhotic patients who underwent LTx with patients who did not undergo LTx was equal to 0.38 (95% CI: 0.17–0.87; *p* = 0.022). In all bivariable models, LTx remained a significant favorable factor for OS (all *p*-values <0.05), with the HR in the same range as in the univariable setting. Also in the multivariable model, cirrhotic patients who received an LTx had a better OS, with a HR equal to 0.25 (95% CI: 0.08–0.74; *p* < 0.01). In multivariable analysis, LTx after surgery had a beneficial impact on both DFS and OS of patients in all the three selection criteria models of LTx (Table 2).

**Table 2:** **Survival after surgery for HCC as function of liver transplant in patients classified within liver transplant criteria**.

	Survival	*N* total	*N* LTx	*N* event	HR	95% CI	*p*-Value
Milan	OS	63	27	26	0.367	0.148–0.914	0.031
	DFS	63	27	43	0.141	0.047–0.421	<0.001
UCSF	OS	68	29	29	0.359	0.151–0.853	0.020
	DFS	68	29	47	0.155	0.057–0.418	<0.001
Up-to-7	OS	68	29	29	0.351	0.148–0.830	0.017
	DFS	68	29	47	0.154	0.057–0.416	<0.001

*LL, UL, lower and upper limit of 95% confidence interval (CI); *N* event, number of events; *N* OLT, number of subjects with LTx; *N* tot, total number of patients; OS, overall survival*.

*Results from a Cox regression with liver transplant (LTx) as a (irreversible) time-dependent factor DFS, disease-free survival; HR, hazard ratio transplant yes vs. no*.

## Discussion

The management of patients with HCC continues to evolve as modern anticancer therapies and improved surgical treatment strategies have translated into better oncologic outcomes. Though, in the absence of high-level evidence to compare results of various surgical therapeutic options, it is not clear which treatment offers the best outcome. Today, LTx seems to be the treatment of choice for early HCC in cirrhotic patients, whereas organ shortage and advances in minimally invasive treatment modalities are challenging the timing and the role of LTx. Although currently OLS is the preferable approach, increasingly more data from specialized centers become available on the safety, feasibility, and even some on the efficacy of minimally invasive liver surgery to treat selected patients with early-stage HCC.

In the current study, we present the results of all consecutive surgically treated patients with HCC from one single institution over a time period of 5 years. A set of 18 variables was taken into consideration to analyze prognostic factors in a cohort of patients with various stages of HCC. We found postoperative LTx as the only independent predictor of both OS and DFS in cirrhotic patients with HCC. Retrospective studies suggest that survival rates for HCC without cirrhosis after LTx are as good as after LR. Though, patients with cirrhosis as an underlying cause of HCC seem to have better outcomes after LTx compared to LR (14–18). However, only a small proportion of patients with cirrhosis is identified in the early-stage of HCC, and thus are candidates within the Milan criteria for LTx. Moreover, lack of donor organ is another major reason of ineligibility. Consequently, LR remains an important therapeutic option in cirrhotic and non-cirrhotic patients with HCC and well-preserved liver function.

The role of LR as bridging therapy for secondary LTx has also been evaluated. The fundamental idea for bridging therapy is to intercept tumor progression and thus, dropout of the transplantation list by LR as waiting times for LTx are long because of organ shortage and LR can be performed without delay. Several studies suggested the feasibility of this strategy and found no difference in morbidity or long-term survival between primary and secondary (after initial LR) LTx (19–21). Laparoscopically performed bridging LR may hereby facilitate the secondary LTx in terms of reduced operative time, blood loss, and transfusion needs (22). In contrast, some studies investigating the outcomes of secondary LTx in the case of underlying cirrhosis suggested the negative impact of this strategy on transplant ability, DFS, and OS (23, 24). In the present study, patients who underwent surgical treatment for HCC within the Milan criteria and subsequently received an LTx had a better survival compared to patients who where treated with surgery alone. LTx offers not only a treatment for the tumor but simultaneously cures the underlying disease, preventing late recurrence. Indeed, in cirrhotic patients with HCC, we found postoperative LTx as the only independent predictor of survival. Another advantage of LR is that it can serve as a selection tool for LTx. Patients with an unfavorable tumor profile and thus high recurrence risk, are preferably treated by other modalities than LTx. Indeed, analysis of strong pathologic predictive factors for recurrence like microvascular invasion or poor histological differentiation can only be performed on the tumor specimen (25). Regarding the three selection criteria systems of LTx candidates (Milan, UCSF, up-to-7) in the entire patient population, we found DFS to be better in patients who met these criteria than in patients who did not. OS, however, was best in patients who only met the Milan criteria. On the other hand, the impact of LTx after surgery was beneficial on both DFS and OS of patients in all the three selection criteria models of LTx.

## Conclusion

Liver transplantation after primary surgery seems to offer the best long-term survival for patients suffering from HCC in cirrhosis as well as for them who fulfill the Milan, UCSF, and up-to-7 criteria. However, shortage of organ donors and relatively high dropout rates on the waiting list are major concerns. Novel treatment strategies are urgently needed in order to improve survival of the increasing number of patients, either as a bridging therapy to LTx while waiting on the list for a transplant, or as alternative therapeutic modalities for those who are no candidates for LTx.

## Author Contributions

Halit Topal participated in the study design, helped with data gathering, and drafted the manuscript. Joyce Tiek participated in the design of the study and gathered raw data. Steffen Fieuws performed the statistical analysis and helped to draft the manuscript. Jacques Pirenne and Frederik Nevens participated in the design of the study, and helped to draft the manuscript. Baki Topal conceived of the study, participated in its design and coordination, and helped to draft the manuscript. All authors read and approved the final manuscript.

## Conflict of Interest Statement

The authors declare that the research was conducted in the absence of any commercial or financial relationships that could be construed as a potential conflict of interest.

## References

[1] World Cancer Report. International Agency for Research on Cancer. Lyon: WHO (2014).

[2] Llovet JM Bruix J Molecular targeted therapies in hepatocellular carcinoma. Hepatology (2008) 48:1312–27 10.1002/hep.22506 18821591 PMC2597642

[3] Llovet JM Burroughs A Bruix J. Hepatocellular carcinoma. Lancet (2003) 362:1907–17. 10.1016/S0140-6736(03)14964-1 14667750

[4] Llovet JM Ricci S Mazzaferro V Hilgard P Gane E Blanc JF Sorafenib in advanced hepatocellular carcinoma. N Engl J Med (2008) 359:378–90 10.1056/NEJMoa0708857 18650514

[5] Huang J Yan L Cheng Z Wu H Du L Wang J A randomized trial comparing radiofrequency ablation and surgical resection for HCC conforming to the Milan criteria. Ann Surg (2010) 252:903–12 10.1097/SLA.0b013e3181efc656 21107100

[6] Bruix J Sherman M Management of hepatocellular carcinoma. Hepatology (2005) 42:1208–36 10.1002/hep.20933 16250051

[7] Livraghi T Meloni F Di Stasi M Rolle E Solbiati L Tinelli C Sustained complete response and complication rates after radiofrequency ablation of very early hepatocellular carcinoma in cirrhosis: is resection still the treatment of choice? Hepatology (2008) 47:82–9. 10.1002/hep.21933 18008357

[8] N’Kontchou G Mahamoudi A Aout M Ganne-Carrié N Grando V Coderc E Radiofrequency ablation of hepatocellular carcinoma: long-term results and prognostic factors in 235 Western patients with cirrhosis. Hepatology (2009) 50:1475–83. 10.1002/hep.23181 19731239

[9] Chen MS Li JQ Zheng Y Guo RP Liang HH Zhang YQ A prospective randomized trial comparing percutaneous local ablative therapy and partial hepatectomy for small hepatocellular carcinoma. Ann Surg (2006) 243:321–8. 10.1097/01.sla.0000201480.65519.b8 16495695 PMC1448947

[10] Mazzaferro V Regalia E Doci R Andreola S Pulvirenti A Bozzetti F Liver transplantation for the treatment of small hepatocellular carcinomas in patients with cirrhosis. N Engl J Med (1996) 334:693–700. 10.1056/NEJM199603143341104 8594428

[11] Mazzaferro V Llovet JM Miceli R Bhoori S Schiavo M Mariani L Predicting survival after liver transplantation inpatients with hepatocellular carcinoma beyond the Milan criteria: a retrospective, exploratory analysis. Lancet Oncol (2009) 10:35–43. 10.1016/S1470-2045(08)70284-5 19058754

[12] Duffy JP Vardanian A Benjamin E Watson M Farmer DG Ghobrial RM Liver transplantation criteria for hepatocellular carcinoma should be expanded: a 22-year experience with 467 patients at UCLA. Ann Surg (2007) 246:502–9. 10.1097/SLA.0b013e318148c704 17717454 PMC1959350

[13] Dindo D Demartines N Clavien PA. Classification of surgical complications: a new proposal with evaluation in a cohort of 6336 patients and results of a survey. Ann Surg (2004) 240:214–5. 10.1097/01.sla.0000133083.54934.ae 15273542 PMC1360123

[14] Iwatsuki S Starzl TE Sheahan DG Yokoyama I Demetris AJ Todo S Hepatocellular carcinoma: comparison between liver transplantation, resective surgery, ethanol injection, and chemoembolization. Ann Surg (1991) 214:221–8. 10.1097/00000658-199109000-00005 1656903 PMC1358637

[15] Colella G Bottelli R De Carlis L Sansalone CV Rondinara GF Alberti A Liver resection versus transplantation for hepatocellular carcinoma in cirrhotic patients. Transpl Int (1998) 11(Suppl 1):S193 10.1007/s001470050459 9664977

[16] Bismuth H Chiche L Adam R Castaing D Diamond T Dennison A. Survival and recurrence after liver transplantation versus liver resection for hepatocellular carcinoma: a retrospective analysis. Ann Surg (1993) 218:145–51. 10.1097/00000658-199308000-00005 8393649 PMC1242923

[17] Otto G Heuschen U Hofmann WJ Krumm G Hinz U Herfarth C Surgical treatment of hepatocellular carcinoma: experience with liver resection and transplantation in 198 patients. Ann Surg (1998) 227:424–32 10.1097/00000658-199803000-00015 9527066 PMC1191281

[18] Ringe B Pichlmayr R Wittekind C Tusch G. Surgical treatment of hepatocellular carcinoma: experience with liver resection and transplantation in 198 patients. World J Surg (1991) 15:270–85. 10.1007/BF01659064 1851588

[19] Kim BW Park YK Kim YB Wang HJ Kim MW. Salvage liver transplantation for recurrent hepatocellular carcinoma after liver resection: feasibility of the Milan criteria and operative risk. Transplant Proc (2008) 40:3558–61. 10.1016/j.transproceed.2008.03.175 19100437

[20] Belghiti J Carr BI Greig PD Lencioni R Poon RT Treatment before liver transplantation for HCC. Ann Surg Oncol (2008) 15:993–1000 10.1245/s10434-007-9787-8 18236111

[21] Belghiti J Cortes A Abdalla EK Régimbeau JM Prakash K Durand F Resection prior to liver transplantation for hepatocellular carcinoma. Ann Surg (2003) 238:885–92. 10.1097/01.sla.0000098621.74851.65 14631225 PMC1356170

[22] Laurent A Tayar C Andréoletti M Lauzet JY Merle JC Cherqui D. Laparoscopic liver resection facilitates salvage liver transplantation for hepatocellular carcinoma. J Hepatobiliary Pancreat Surg (2009) 16:310–4. 10.1007/s00534-009-0063-0 19280110

[23] Poon RT Fan ST Lo CM Liu CL Wong J. Long-term survival and pattern of recurrence after resection of small hepatocellular carcinoma in patients with preserved liver function: implications for a strategy of salvage transplantation. Ann Surg (2002) 235:373–82. 10.1097/00000658-200203000-00009 11882759 PMC1422443

[24] Adam R Azoulay D Castaing D Eshkenazy R Pascal G Hashizume K Liver resection as a bridge to transplantation for hepatocellular carcinoma on cirrhosis: a reasonable strategy? Ann Surg (2003) 238:508–19. 10.1097/01.sla.0000090449.87109.44 14530722 PMC1360109

[25] Forner A Llovet JM Bruix J. Hepatocellular carcinoma. Lancet (2012) 379:1245–55. 10.1016/S0140-6736(11)61347-0 22353262 PMC13537732

